# Development and Evaluation of Inhalational Liposomal System of Budesonide for Better Management of Asthma

**DOI:** 10.4103/0250-474X.73916

**Published:** 2010

**Authors:** J. J. Parmar, D. J. Singh, Darshana D. Hegde, A. A. Lohade, P. S. Soni, A. Samad, Mala D. Menon

**Affiliations:** Bombay College of Pharmacy, Kalina, Santacruz (East), Mumbai-400 098, India; 1 Board of Radiation and Isotope Technology and Medical Cyclotron Facility Parel, Mumbai-400012, India; 2 Department of Medicine, Bombay Veterinary College, Parel, Mumbai-400 012, India

**Keywords:** Budesonide, liposome, inhalation, pulmonary delivery, twin stage impinger

## Abstract

Budesonide is a corticosteroid used by inhalation in the prophylactic management of asthma. However, frequent dosing and adverse effects (local and systemic) remain a major concern in the use of budesonide. Liposomal systems for sustained pulmonary drug delivery have been particularly attractive because of their compatibility with lung surfactant components. In the present investigation, pulmonary liposomal delivery system of budesonide was prepared by film hydration method and evaluated for sustained release. Various parameters were optimized with respect to entrapment efficiency as well as particle size of budesonide liposomes. For better shelf life of budesonide liposomes, they were freeze dried using trehalose as cryoprotectant. The liposomes were characterized for entrapment efficiency, particle size, and surface topography; *in vitro* drug release was evaluated out in simulated lung fluid at 37° at pH 7.4. The respirable or fine particle fraction was determined by using twin stage impinger. The stability study of freeze dried as well as aqueous liposomal systems was carried out at 2-8° and at ambient temperature (28±40). The freeze dried liposomes showed better fine particle fraction and drug content over the period of six months at ambient as well as at 2-8° storage condition compared to aqueous dispersion of liposomes.

Inhalation delivery of medications in the treatment of lung diseases like asthma, lung cancer and COPD is advantageous over the conventional systemic therapy as it is a non-invasive mode of administration that avoids first pass metabolism along with reduction in systemic side effect. Although this mode is an effective means for delivering relatively small quantities of drugs to target sites, several drugs are rapidly cleared from the lungs and pass into the systemic circulation, which necessitates frequent dosing and the occurrence of unwanted systemic side effects. In addition, this leads to poor patient compliance to therapeutic regime and the risk of associated adverse effects increases. In such cases, sustained release of locally acting drugs in the lung would be particularly beneficial, since they could be delivered to and retained at the targeted receptors for prolonged period of time and thus minimize the biodistribution throughout the systemic circulation[1–7].

Various methods have been investigated to achieve pulmonary sustained release systems for short-acting drugs, like incorporation of drugs in liposomes and other biodegradable microspheres, the modification of chemical structure to produce either prodrugs or drug conjugates with macromolecules and complexes of the drug with cyclodextrins. Of these, liposomes have received a lot of attention.

Liposomes offer several attractive features for pulmonary drug delivery. They can serve as a solubilization matrix for poorly soluble agents, act as a pulmonary sustained release reservoir, facilitate intracellular delivery of drugs, specifically to alveolar macrophages, show good compatibility with lung surfactant components, low order of local irritation to lung tissue and hence reduced pulmonary toxicity[3,8,9].

The high safety margin of inhaled corticosteroids (compared to the oral route) has encouraged their widespread use in patients with moderate to severe asthma. Frequent dosing requirement and local and systemic adverse effects remain a major concern for such inhaled corticosteroids[10]. Budesonide (BDS) is a corticosteroid used in the prophylactic management of asthma. Currently BDS formulations are available as immediate release aerosol systems, which need repeated administration.

Sustained release formulation of BDS in the lung can offer prolonged retention time as well as minimized biodistribution throughout the systemic circulation. Furthermore, it will be the ideal treatment effective in preventing bronchospasm for 6-8 h during which the patients are asleep[11]. Thus, there is a need to develop sustained release budesonide formulations that can prolong the drug release and hence, the antiinflammatory action[12,13]. The objective of the present work was to develop an effective sustained action BDS liposomal aerosol formulation for lung delivery, and thereby achieve improved therapy in asthma.

## MATERIALS AND METHODS

Phospholipon 90 G and Phospholipon 90 H were kindly gifted by Natterman GmbH Germany. Budesonide (BDS) gifted by Cipla Ltd. Mumbai, India; while trehalose was a kind gift from Hyashibara, Japan. Cholesterol and all the other solvents and chemicals of analytical grade were obtained from S. D. Fine-Chem, Mumbai, India.

### Preparation of BDS-liposomes:

Liposomes were prepared by lipid film hydration method[14]. The hydration of the lipid film was carried out with hydroxypropyl-β-cyclodextrin (HPβCD) solution at 60°. The formed liposomes were allowed to anneal by heating (60° for 10 min) in a water bath (Superfit, India). Vesicle size was reduced by subjecting the liposomes to mechanical shaking on a horizontal shaker bath (Expo, India) for up to 6 h with intermittent sonication (30 s), carried out after every two hours, in a bath sonicator (Expo, India) at room temperature. The unentrapped drug was removed by centrifugation (20 000 g, Beckman, Alaska, USA). The liposomal dispersion of BDS was freeze dried using trehalose as cryoprotectant (Labconco, Kansas city, USA), and they were stored in a refrigerator in sealed vials until use. Thermal behaviour of BDS before and after freeze drying was studied using DSC (DT40, Shimadzu Tokyo, Japan).

### Characterization of freeze dried liposomes:

The particle size distribution of the liposomes was assessed on a Malvern Mastersizer (Malvern Instruments, Boston, USA) after rehydration with saline. Transmission electron microscopy (TEM) images were visualized using the electron microscope (Jeol JEM-1010, Japan) and photographed. Surface topography of the freeze dried liposomes was determined using environmental scanning electron microscope (Quanta-200, FEI Hillsboro OR, USA). To observe the reformation of liposomes in presence of saline, the dry powder was wetted with a drop of saline, and observed over a period of 5 min under ESEM and photographed.

### Determination of entrapment efficiency:

The Determination of entrapment efficiency was determined using reverse phase HPLC. The chromatographic system consisted of Jasco PU 980 Intelligent Pump, Jasco UV 975, a UV/Vis detector Version 1.50 Build 15 (Jasco-BorwinTM, Tokyo, Japan) and Rheodyne injector valve bracket fitted with a 20 µl sample loop. HPLC separations were performed on a stainless-steel Technochrom kromacil; C-18 analytical column (250×4.6 mm) packed with 5 µm diameter particles. Data was processed using Chromatography Software, Hercule 2000 chromatography interface star 800 interface module interface Version 2.0 (Jasco-Borwin^™^, Tokyo, Japan). The composition of the mobile phase was methanol:glacial acetic acid 0.1%, (69:31). The mobile phase was delivered at a flow rate of 1 ml/min. The injection volume was 20 µl and the retention time was found to be 5.0 min.

For EE of BDS liposome, 0.25 ml of liposomal preparation was diluted to 4 ml with distilled water and centrifuged at 20 000 g at 8°±0.5° for 30 min using Allegra^™^ 64 R centrifuge (Beckman, USA). The supernatant was appropriately diluted in distilled water and the amount of drug present in the supernatant was determined by reverse phase HPLC. Entrapment efficiency was calculated as the difference between the initial amount of drug added and that present in the supernatant as free drug, and was expressed as µg of drug entrapped per mM of total phospholipids (µg/mM of lipid).

### *In vitro* drug release:

*In vitro* drug release profile of BDS from freeze dried liposomes was assessed in simulated lung fluid (pH 7.4) at 37° by static method. After periodic intervals of time, the liposomal aliquots were centrifuged and supernatant evaluated for the drug content by HPLC.

### *In vitro* drug deposition studies by Twin Stage Impinger (TSI):

The respirable fine particle fraction (FPF) of the BDS liposomes was determined using a twin-stage impinger- apparatus A (TSI), official in British Pharmacopoeia. The liposomal dispersion prepared by reconstitution in saline was nebulized using the nebulizer (Omron CX3) attached to the TSI by means of rubber collars. The vacuum pump was operated at 28 l/min. The fractions collected in each stage were analyzed for the drug content by HPLC using the chromatographic conditions as described above.

### Stability studies:

Stability evaluation of aqueous liposome suspension and freeze dried liposomes was carried out at 2-8° and ambient condition (28±4°) for a period of six months. Samples withdrawn at specified time intervals (1, 3 and 6 mo) were evaluated for the drug content, particle size and FPF. Also the liposomes were visually observed for appearance, ease of reconstitution, and sedimentation.

## RESULTS AND DISCUSSION

One of the perceived benefits of liposomes as drug carrier is based on their ability to alter favuorably the pharmacokinetic profile of the encapsulated species and thus provide selective and prolonged pharmacological effects at the site of administration. BDS, being practically insoluble in water, may precipitate out on hydration of liposomes. This would lower the entrapment efficiency and possibly destabilize the liposomes. Many studies report the use of HPβCD drug complexes to improve the entrapment and impart stability to liposomes[15,16]. To determine the effect of hydration medium on entrapment efficiency of BDS, liposomes were prepared by lipid film hydration using distilled water or as well as HPβCD solution; varying concentrations of HPβCD solutions (0, 3, 6, 9, 12, 15, 21 mM) were tried as hydration medium. The solubility diagram was indicative of type A_L_ complex. Since, the slope of the line is less than 1, the complex stoichiometry was assumed to be 1:1 and the apparent stability constant K_1:1_ was found to be, 938.37 M^-1^. The HPβCD as hydration medium increased the entrapment efficiency of BDS by 50%. This improved entrapment efficiency can be attributed to this complexation. The solution of BDS complexed HPβCD is able to travel inside the liposomes during hydration and lead to increase in entrapment efficiency.

Entrapment efficiency depends on the formulation variables. To study this aspect, several batches of BDS liposomes were prepared with varying lipid compositions; the EE, composition and particle size are mentioned in Table 1. Decreased EE as after increasing cholesterol may be attributed to the lipophilic nature of cholesterol which could possibly be competing with BDS in the lipid bilayer and reducing the entrapment of BDS. Glavas-Dodov *et al*. have reported similar observations for 5-flourouracil[17].

**TABLE 1:** EFFECT OF FREEZE DRYING ON DRUG CONTENT AND PARTICLE SIZE

Batch code	Composition			Drug content (µg/mM of lipid)		Mean particle size (µm)	
	CH	PL 90 G	PL 90 H	Before freeze drying	After freeze drying	Before freeze drying	After freeze drying
BL-1	2	1	9	6037.18±289.30	6097.66±174.28	4.98	6.12
BL-4	2	3	7	5324.24±66.07	5238.64±210.20	4.20	6.17
BL-7	2	5	5	4669.64±157.18	4813.59±89.48	3.97	6.28
BL-10	6	1	9	2842.53±262.39	2950.89±195.07	6.47	7.29
BL-13	6	3	7	2580.20±114.12	2786.99±87.54	4.50	7.18
BL-16	6	5	5	2590.80±223.51	2820.34±118.20	5.47	6.74
BL-19	10	1	9	2200.42±149.00	2398.61±169.05	6.47	7.85
BL-22	10	3	7	2525.54±139.05	2699.29±153.29	6.27	7.49
BL-25	10	5	5	2440.53±80.84	2614.17±121.55	6.58	7.95

Increasing the amount of Phospholipon 90 G increased the EE of BDS. This could be attributed to the fact that unsaturated phospholipids are relatively more flexible in nature, and thereby provide less hindrance for BDS to be retained in lipid bilayer. Increasing the saturated lipid, Phospholipon 90 H resulted in reduced EE; this could be due to the saturated lipid behaving in the same way as cholesterol. Better hydration was achieved by increasing shaking time leading to increased EE, which could be attributed to better solubilization of BDS in HPβCD.

Liposomal delivery system is susceptible to degradation. Various approaches have been developed and evaluated to impart long term stability to liposomes and one of them is lyophilization[18]. To maintain the same particle size distribution and to avoid leakage of the encapsulated drug from liposomes after the freeze-drying/rehydration cycle, suitable cryoprotectant like disaccharides need to be added, which prevent fusion, aggregation and leakage of encapsulated compounds[17]. In the present study, liposomes were freeze dried using various ratios of lipid: cryoprotectants. Different disaccharides like lactose, mannitol, maltodextrin, and trehalose were evaluated for their cryoprotective effects at varying ratios. Lipid:trehalose at a ratio of 1:10 was found to be most suitable, as less drug leakage and minimal change of particle size were observed after freeze drying.

**Fig. 1:**
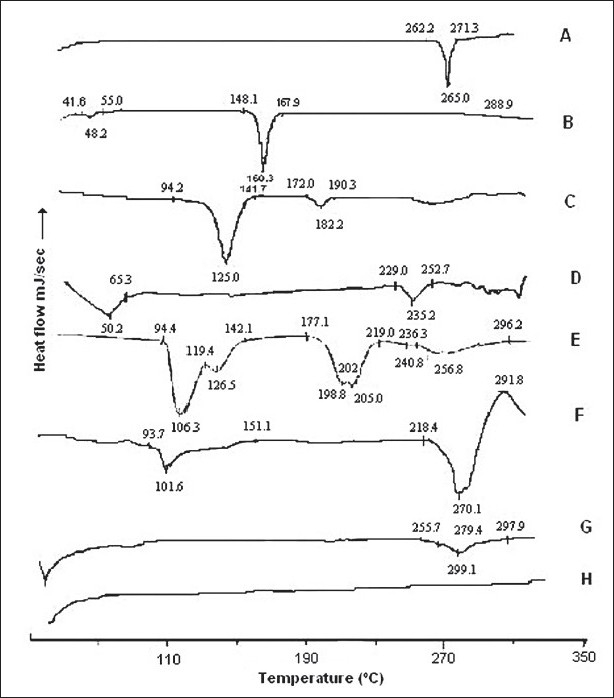
DSC thermograms DSC thermograms for (a) budesonide, (b) cholesterol, (c) PG90H, (d) PG90G, (e) trehalose, (f) freeze dried liposomes, (g) freeze dried liposomes without trehalose, (h) blank liposomes without trehalose, which show there is interaction of trehalose with phospholipids of liposomes during freeze drying

DSC spectra of BDS in fig. 1 showed one sharp endotherm at 265.8° with onset temperature 262.3° and recovery temperature 271.3°. Freeze dried liposomes gave thermal events in the range of 93.7-151.1°, and a big endothermic peak at 270.1° immediately followed by exotherm with peak temperature at 291.8°. This event indicated the interaction of trehalose with phospholipids of liposomes during freeze drying.

The sharp endotherm of budesonide at 265.8° was masked when freeze dried BDS-loaded liposomes were analyzed. The broad endotherm started at 248.4° with peak of endotherm at 270.1° and followed immediately by exotherm at 291.8°. So to detect the presence of BDS in liposome, a DSC scan was carried out on freeze dried blank and BDS-loaded liposomes without trehalose. Blank liposomes did not show any thermal event for the entire temperature range studied, while a small broad endotherm was observed at 269.1° in BDS loaded liposomes indicating presence of the drug. The shift in the endotherm from 265.8° may be due to the presence of lipids

**Fig. 2:**
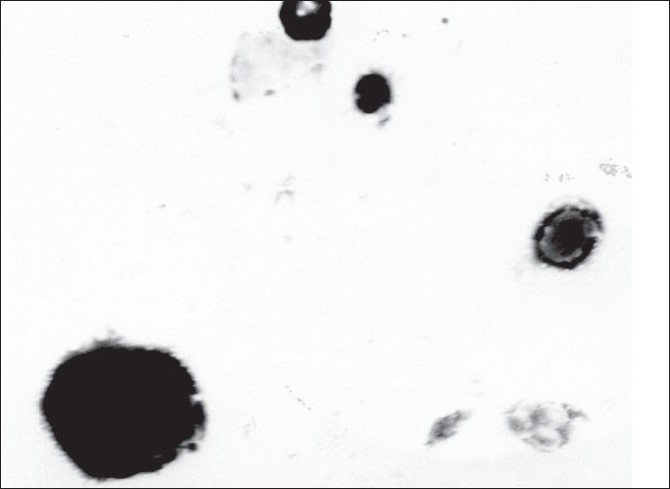
TEM photomicrograph of BDS liposomes TEM images of BDS loaded liposomes appearing as bright spheres surrounded by dark thick layer showing large internal aqueous core

**Fig. 3:**
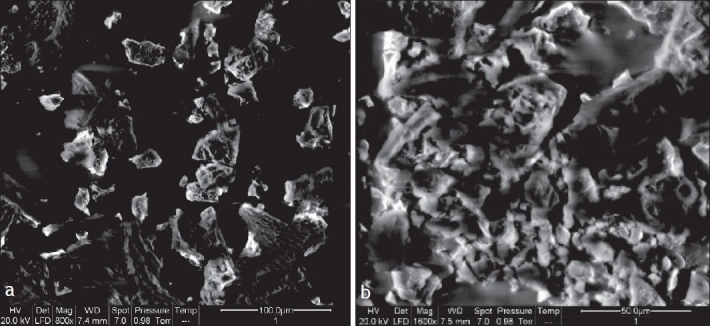
ESEM photomicrograph of freeze dried trehalose particles ESEM photomicrograph shows that freeze dried trehalose particles (a) were of irregular porous particles and freeze dried liposomes (b) appeared as aggregated particles with lipids on the surface

The TEM images of BDS loaded liposomes are shown in fig. 2. The liposomes appeared as bright spheres surrounded by dark thick layer showing large internal aqueous core. The ESEM analysis showed the freeze dried trehalose particles as irregular porous particles as shown in fig. 3a. However, the freeze dried liposomes fig. 3b appeared as aggregated particles with lipids on the surface.

**Fig. 4:**
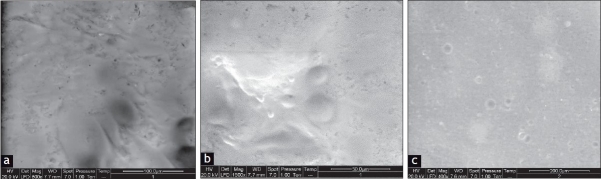
ESEM photomicrograph of freeze dried BDS liposome hydrated with saline ESEM photomicrograph of freeze dried BDS liposome hydrated with saline shows initiation of formation of liposome at 0 min (a), further at 2 min there was sufficient hydration of lipids and spherical structures indicates complete formation of liposomes (b), and finally at 5 min entire area was filled with formed liposomes (c)

ESEM was used to study liposome formation from freeze dried liposomes and to provide an alternative method to observe liposome formation after rehydration. Dynamic formation of liposomes was monitored by placing drop of saline on the tab and analyzing over a period of 5 min. The ESEM photographs of hydrated freeze dried liposomes at 0, 2 min and after complete hydration (5 min) are shown in figs. 4a–c, respectively. At 0 min, after adding few drops of saline on tab with freeze dried liposomes, trehalose started dissolving in saline and liposome formation was initiated due to budding off mechanism. Following sufficient hydration of the lipids on trehalose particles spherical structures were clearly seen, after two minutes liposomes were completely formed and after 5 min entire area was filled with formed liposomes.

The particle size increased in all the liposomes irrespective of the formulation variables indicating aggregation of liposomes after freeze drying. However, no significant difference was observed in drug content after freeze drying of BDS liposomes indicating minimum leakage. Table 2 shows the result of drug content and particle size before and after freeze drying. These results indicate the protection provided by trehalose.

**TABLE 2:** DRUG LEAKAGE AND PARTICLE SIZE OF STABILITY SAMPLES OF BDS LIPOSOMES

	Aq. dispersion (After six month)			Freeze dried liposome (After six month)		
	Initial	2-8°	Ambient	Initial	2-8°	Ambient
Mean particle Size (µ)	4.56±0.25	14.45±1.21	21.22±0.64	6.31±0.41	7.89±0.74	8.43±0.91
Drug leakage (%)	3.23±1.02	17.58±2.58	30.07±2.60	1.03±0.90	8.38±1.49	6.30±1.47
Fine particle fraction (FPF) (%)	48.69±1.86	35.18±3.72	28.17±3.84	42.29±2.84	41.28±3.17	40.48±1.91

**Fig. 5:**
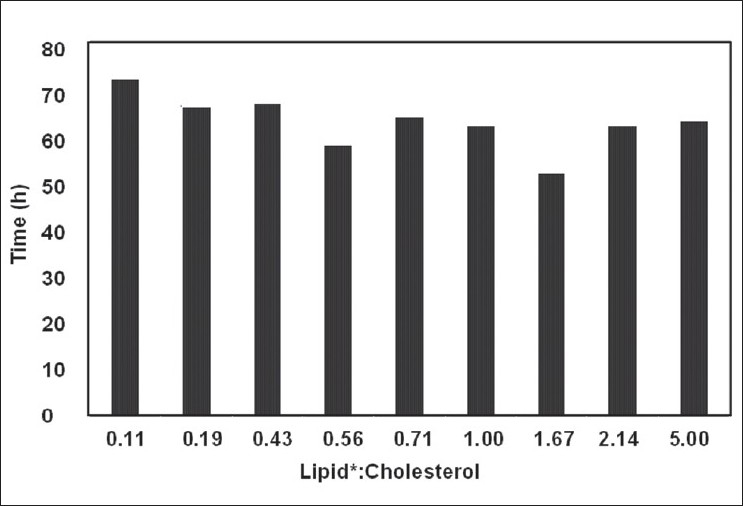
Time for 80% drug release from BDS liposomes It shows the time required for 80% drug release (T_80%_) with respect to formulation variables, and plotted against the ratio of lipid (90G:90H):cholesterol

The drug release was sustained for more than 70 h for all batches but there was significant difference in release rate of the drug between the batches as shown in fig. 5. The formulation variables such as cholesterol content, 90 G and 90 H, played a major role in the drug release. Fig. 5 shows the time required for 80% drug release (T_80%_) with respect to formulation variables, and plotted against the ratio of lipid (90G:90H):cholesterol. It clearly showed that increase in the cholesterol content reduced T_80%_. Increase in 90 G decreased T_80%_ while increase in 90 H increased T_80%_. Thus it can be concluded that, the permeability of the bilayer is strongly influenced by its constituents. It can be seen that high amount of cholesterol and 90 H significantly reduced the T_80%_, indicating increased rigidity of bilayer

Stability of freeze dried liposomes and aqueous dispersion of BDS liposomes was assessed at 2-8° and at ambient conditions (28±4°). Stored samples were evaluated periodically for change in colour, appearance, particle size and drug leakage. The change in particle size as well as drug leakage from the freeze dried liposomes and aqueous suspension of liposomes in stability studies are as given in Table 2.

Sedimentation of lipid phase was observed in aqueous liposomes, which was easily redispersed by gentle shaking, and no colour change was evident upon storage. Microscopic observation of aqueous liposomes showed absence of drug precipitation; however the particle size was markedly increased.

At 2-8°, aqueous liposomes showed lesser aggregates compared to the ones stored at ambient conditions in which aggregates of liposomal vesicles were observed. Particle size distribution of aqueous liposomes stored at different conditions showed a shift towards increased particle size as well as its range, with time. Several light scattering studies of the coalescence kinetics of liposomes have revealed that the average cluster size grows with time following a characteristic behaviour. After the aggregation, liposomes tend to form large vesicles via coalescence[3].

Liposomes were easily formed when stored samples of freeze dried liposomes, was mixed with saline on gentle shaking, and no color change was seen upon storage. Microscopic observation of rehydrated freeze dried liposomes did not show drug crystals. No significant difference was observed in the particle size of freeze dried liposomes stored for a period of six months at different conditions.

Drug leakage was significantly less in freeze dried liposomes compared to aqueous liposomes. The drug leakage was in accordance with the increase in particle size. It was observed that increase in particle size was directly associated with drug leakage.

The effect on FPF of the freshly prepared liposomes was compared with stored freeze dried and aqueous liposomes. FPF was determined using TSI. In case of freeze dried liposomes, no significant change in FPF was seen at both the storage conditions, over a period of six months, whereas aqueous liposomal dispersions showed decrease in FPF at the different storage conditions. This may be attributed to the increase in particle size, due to aggregation.

Drug delivery to or via the respiratory tree has been a long standing pharmaceutical objective. For locally acting agents it is desirable to confine the action of the drug to the lungs in order to eliminate unintended side effects, which might result following absorption and distribution to other extra vascular sites. Oral inhalation is often the preferred route in order that such effects can be minimized. In the present investigation, BDS-loaded liposomes were developed optimized and evaluated for its suitability for pulmonary sustained drug delivery. Liposomes were successfully developed and freeze dried using trehalose as cryoprotectant. Further *in vivo* safety and deposition studies in suitable animal models will give better insight for clinical applications of the inhalable BDS liposomes.
